# Exploring the impact of immersive virtual reality on depression knowledge and stigma reduction: a cross-over RCT fMRI study

**DOI:** 10.1038/s41598-024-55797-w

**Published:** 2024-03-02

**Authors:** Wey Guan Lem, Kelssy Hitomi dos Santos Kawata, Hiroshi Oyama

**Affiliations:** Department of Clinical Information Engineering, Graduate School of Medicine, The University of Tokyo, Hongo 7-3-1, Bunkyo-ku, Tokyo, 113-0033 Japan

**Keywords:** Public health, Psychology

## Abstract

The stigma of mental illness is a form of negative judgmental knowledge and is a barrier to individual seeking treatment. Contact-based educational interventions with first-person perspective (1PP) combined with immersive virtual reality (IVR) is promising as an anti-stigma intervention. This study aims to investigate the effectiveness of 1PP anti-stigma IVR intervention compared to video in enhancing depression knowledge and reducing stigma, as well as to examine the corresponding depression knowledge brain activity change using functional magnetic resonance imaging (fMRI). Participants engaged in a 1PP anti-stigma intervention using both IVR and conventional video, focusing on the daily life and recovery of a patient with mild depression. The change in depression knowledge, stigma-related behavioral, and brain activity using fMRI were measured at pre- and post-interventions. Depression knowledge improved for both interventions; however, only the IVR intervention reduced stigma. In the IVR intervention, depression knowledge score was positively associated with neural response in the right superior frontal gyrus activation, indicative of empathic concern. Conversely, the video intervention correlated with increased activity in the right anterior insula, suggesting a distress-related response. The findings demonstrate that the immersive nature of IVR can reduce stigma more effectively than video intervention. This effectiveness is underpinned by the change in depression knowledge on neural activity, with IVR fostering empathy-related behavioral responses. The results highlight the potential of IVR in enhancing empathic understanding and reducing stigma towards mental illness, emphasizing the need for further exploration into immersive technologies for mental health education.

## Introduction

Stigma, characterized by discrimination, prejudice, and stereotypes, particularly impacts minority groups such as individuals with mental illnesses^1^. The stigma of mental illness is a significant issue as it is a form of negative judgmental knowledge, which impedes patients’ treatment, leading to societal challenges such as unemployment, especially in cases of depression^2,3^. Studies have shown that inaccurate knowledge about mental illness can provoke multisensory aversive feelings, such as disgust, fear, or anger, leading to discriminatory behavior^1,4^. Interestingly, stigmatizing attitudes vary with individual knowledge about mental illness^5,6^.

Contact-based educational interventions employing a first-person perspective (1PP) are increasingly recognized for their effectiveness in reducing stigma^7,8^. Contact-based educational interventions typically involve disseminating accurate mental illness information^8,9^, patient testimonials^7–9^, and successful stories on recovery^10,11^. The three key mediators in such interventions are knowledge gain, anxiety reduction, and adopting a 1PP^12^. The 1PP is particularly impactful, allowing individuals without mental illness to empathically understand the internal states of those with mental illness, thus mitigating stigma^12^. However, its effectiveness depends on the individual’s prior mental illness knowledge to use their imagination to understand individuals with mental illness (outgroup perspective)^13,14^.

Recent studies have explored using immersive virtual reality (IVR) technology for 1PP interventions to reduce stigma^15,16^. IVR, utilizing a head-mounted display (HMD), enhances the sense of presence (“being there”) and embodiment in a realistic, multisensory environment, potentially more effective than traditional video methods^13,17–19^. Enhanced presence and embodiment in IVR, achieved through immersive storytelling^20^, avoidance of cognitive overload^21^, and interaction with a 1PP avatar^22^, showing recovery^15,16^, have been shown to influence empathy and knowledge retention. However, the effectiveness of IVR in reducing stigma through synchronized body-tracking interaction remains unverified, and its neural correlates in mental illness knowledge acquisition are unknown.

Neuroimaging studies have suggested the involvement of frontal lobe and anterior insula in stigma and social cognition related processes. The superior frontal gyrus (SFG), primary motor cortex, and supplementary motor cortex are related to the frontal lobe and, together with anterior insula, play important roles in the experience of discrimination, which is one of the core processes of stigma^23^. In addition, the SFG and anterior insula are also involved in the perception experience of empathy, disgust, social rejection, and social exclusion^24–31^. On the other hand, although the SFG and anterior insula are involved in similar types of perception experience generated from the emotion of outgroup individuals, some of these studies showed opposite emotional valence functions. For example, the downward social comparison with an outgroup^27^ and empathic concern felt towards physically ill patients^31^ are related to the SFG activation and considered as a reward stimulus with positive valence. In contrast, racial prejudice experience^28^ and empathic distress felt towards mentally ill patients^31^ are associated with anterior insula activation and considered as negative valence.

In terms of individual variation, neuroimaging studies have explored neural correlates of stigma-related processes by correlational analysis using self-reported measures of negative attitudes scores^24,28,32,33^. They compared groups with different levels of neural activation during manipulation of disgust and social exclusion experience caused by different social minority groups, such as homeless and alcoholic individuals. For the SFG, the more negative implicit attitudes the participants had toward the stigmatized group, the greater their neural activation in the R.SFG in the stigma condition^32^. For the insula, negative explicit attitudes toward homeless individuals were associated with increased activity in the insula^33^. Moreover, increased activation of the anterior insula was associated with social exclusion experience^24^. Furthermore, an individual’s disgust sensitivity can positively predict the activity in the right anterior insula^28^. However, to the best of our knowledge, it is unknown whether the stigma and social cognition-related brain regions can be extended to the 1PP anti-stigma IVR intervention.

**Figure 1:**
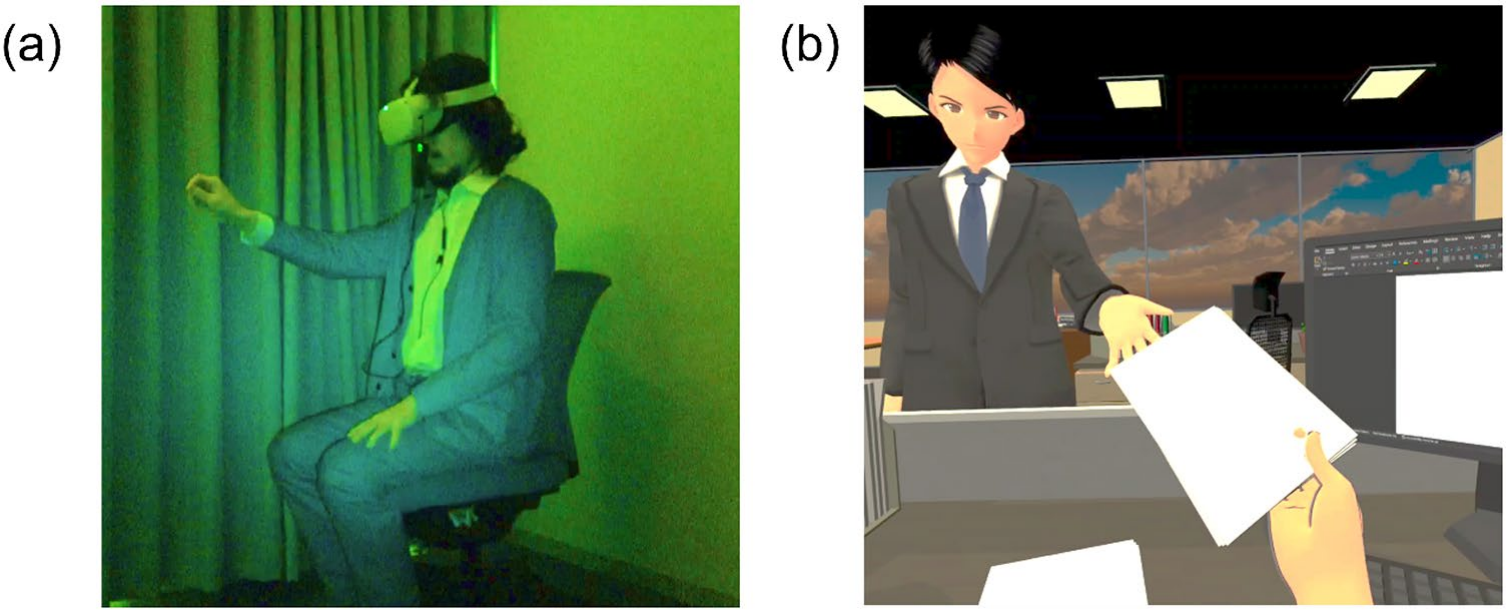
(**a**) Depiction of the setup used for the IVR first-person perspective anti-stigma intervention. (**b**) Screenshot of the content of IVR and video interventions. *IVR* immersive virtual reality.

The current study was designed in response to the limitations of 1PP anti-stigma IVR intervention and the need for relevant bio-psychological studies in the same field. Using the HMD (Fig. 1a); the 1PP anti-stigma IVR intervention immersed participants in the daily routine of a patient with mild depression. The symptoms of mild depression were chosen to be simulated as the mental illness 1PP anti-stigma intervention scenario for three main reasons. First, the symptoms of depression by itself may increase the stigma of the general public towards patients with depression^34^, such as being absent from work using depression as an excuse (i.e. malingering)^34^. Second, mild symptoms of depression were also considered to reduce the risk of participants being affected by the negative emotions or depression portrayed in the 1PP simulation. Third, the IVR simulation of hallucinations for mental illnesses such as Schizophrenia may have the reverse effect of increasing stigma^35^.

We conducted a cross-over randomized control trial (RCT) to investigate the effects of interactive 1PP anti-stigma IVR intervention on depression knowledge, reduction in stigma, and the corresponding change in depression knowledge on brain activity using functional magnetic resonance imaging (fMRI). For that, participants were exposed to both the IVR and video interventions, with video selected for its established role in contact-based educational methods^8,11^. The mental illness and disorder understanding scale (MIDUS)^36^ and the intended behavior subscale of the reported intended behavior scale – Japanese (RIBS-J)^37,38^ were used to assess depression knowledge and stigma, respectively, before and after each intervention. Additionally, we conducted a 1PP anti-stigma auditory fMRI task to minimize the visual effect at post-intervention, followed by assessments of perception of re-experience and embodiment of the interventions^39^.

We hypothesized that the 1PP anti-stigma IVR intervention would be more effective than the video intervention in improving depression knowledge and reducing stigma. As intervention using IVR improves the sense of presence and embodiment experience, we predicted that the individual variation in depression knowledge acquisition after the 1PP IVR intervention would be associated with empathic concern and increase activity in the SFG. On the other hand, video intervention would be associated with distress-related response and increase activity in the anterior insula.

## Results

We initially screened 40 participants for eligibility; of these, 36 participants fulfilled the inclusion criteria and were randomly assigned to the IVR-video sequence intervention group (IVR Day 1 and video Day 2 sequence; 18 participants) and the video-IVR sequence intervention group (video Day 1 and IVR Day 2 sequence; 18 participants). Of the 36 participants, two did not attend Day 2 of the IVR intervention, and two were excluded from the analysis because of excessive head movements (i.e.  > 7 mm) in the MRI scanner. Therefore, 32 participants completed all the assessments, interventions, and fMRI scanning: 18 participants in the IVR-video sequence intervention group and 14 participants in the video-IVR sequence intervention group (Refer to Supplementary Figure S1). There was no statistical difference in terms of demographic characteristics (Table 1), the carryover effect (*p* = 0.27) of the MIDUS score, and the intended behavior subscale of the RIBS-J score (*p* = 0.41) between IVR-video and video-IVR sequence intervention groups. Thus, we analyzed behavioral and brain data from 32 participants (13 female; age range = 19–46 years, age [mean ± standard deviation] = 24.15 ± 6.21 years) for each IVR and video intervention type. The demographic characteristics for the total sample and each sequence intervention group (i.e. IVR-video and video-IVR sequence groups) are shown in Table 1.

**Table 1:** Demographic characteristics [mean (SD)].

	Total sample	IVR-VIDEO	Video-IVR	t/X^2^	*p*-value
Number of participants	32	18	14		–
Age (years)	24.15 (6.21)	23.87 (5.52)	24.51 (7.41)	− 0.28	0.78
Sex (female/male)	13/19	8/10	5/9	0.25	0.62
Contact with depression patient % (yes/no)	28.1/71.9	33.3/66.6	21.4/78.6	0.55	0.46
Learned about depression % (yes/no)	28.1/71.9	27.7/72.3	28.6/71.4	0.01	0.96
IVR experience % (yes/no)	53.1/46.9	55.6/44.4	50.0/50.0	0.10	0.76

*SD* standard deviation*, IVR* immersive virtual reality.

The time spent on the interventions required 8.10 ± 1.28 min (range 7–12) for the 1PP anti-stigma IVR intervention and 6.75 ± 0.72 min (range 5–8) for the video intervention.

### Effect of anti-stigma intervention on depression knowledge-related behavioral changes

**Figure 2:**
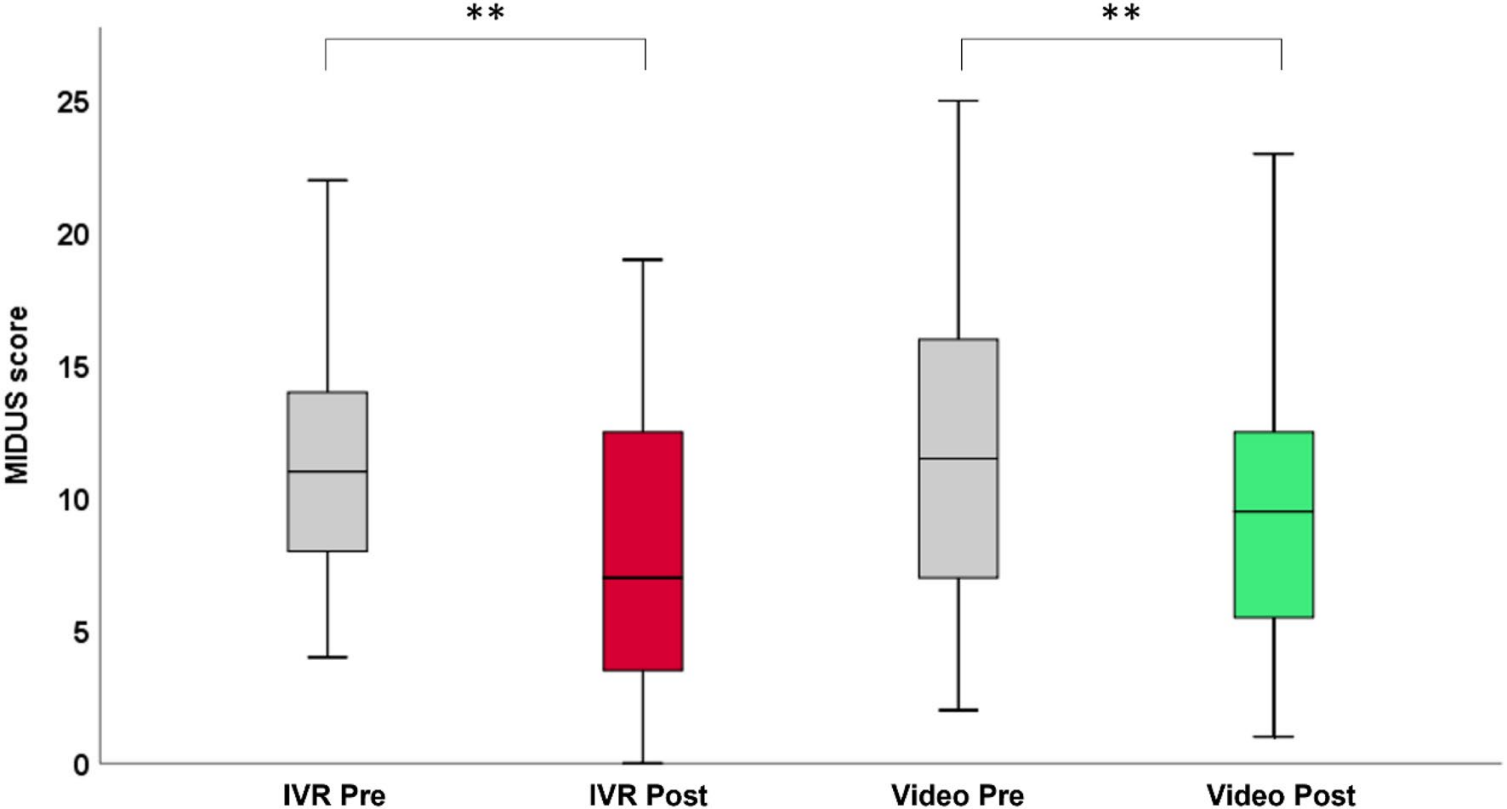
Change in the mean absolute total MIDUS score at pre- and post-interventions for the IVR and video. *IVR* immersive virtual reality, *MIDUS* mental illness and disorder understanding scale. **p < 0.001.

To assess whether anti-stigma intervention with IVR and video interventions affects depression knowledge-related behavioral change, we performed a two-way repeated measures analyses of variance (RM-ANOVA) for mean total MIDUS score with time as a within-subject factor (pre-intervention vs. post-intervention) and intervention type as the between-subject factor (IVR intervention vs. video intervention). The main effect of time showed a statistically significant difference in mean total MIDUS scores across pre- and post-interventions (F (1, 31) = 37.29, *p* < 0.001). The main effect of intervention showed no statistically significant difference in mean total MIDUS scores between IVR and video intervention types (F (1, 31) = 3.03, *p* = 0.09). There was no statistically significant interaction between intervention type and time on mean total MIDUS scores (F (1, 31) = 0.20, *p* = 0.66). Post-hoc analysis with Bonferroni adjustment on the main effect of time revealed that mean total MIDUS scores significantly reduced from pre-intervention to post-intervention in IVR intervention ([mean ± standard deviation] = 11.09 ± 4.39 vs. 8.25 ± 5.19, *p* < 0.001) and video intervention ([mean ± standard deviation] = 11.94 ± 5.63 vs. 9.47 ± 5.45, *p* < 0.001) (Fig. 2).

### Effect of anti-stigma intervention on stigma-related behavioral changes

**Figure 3:**
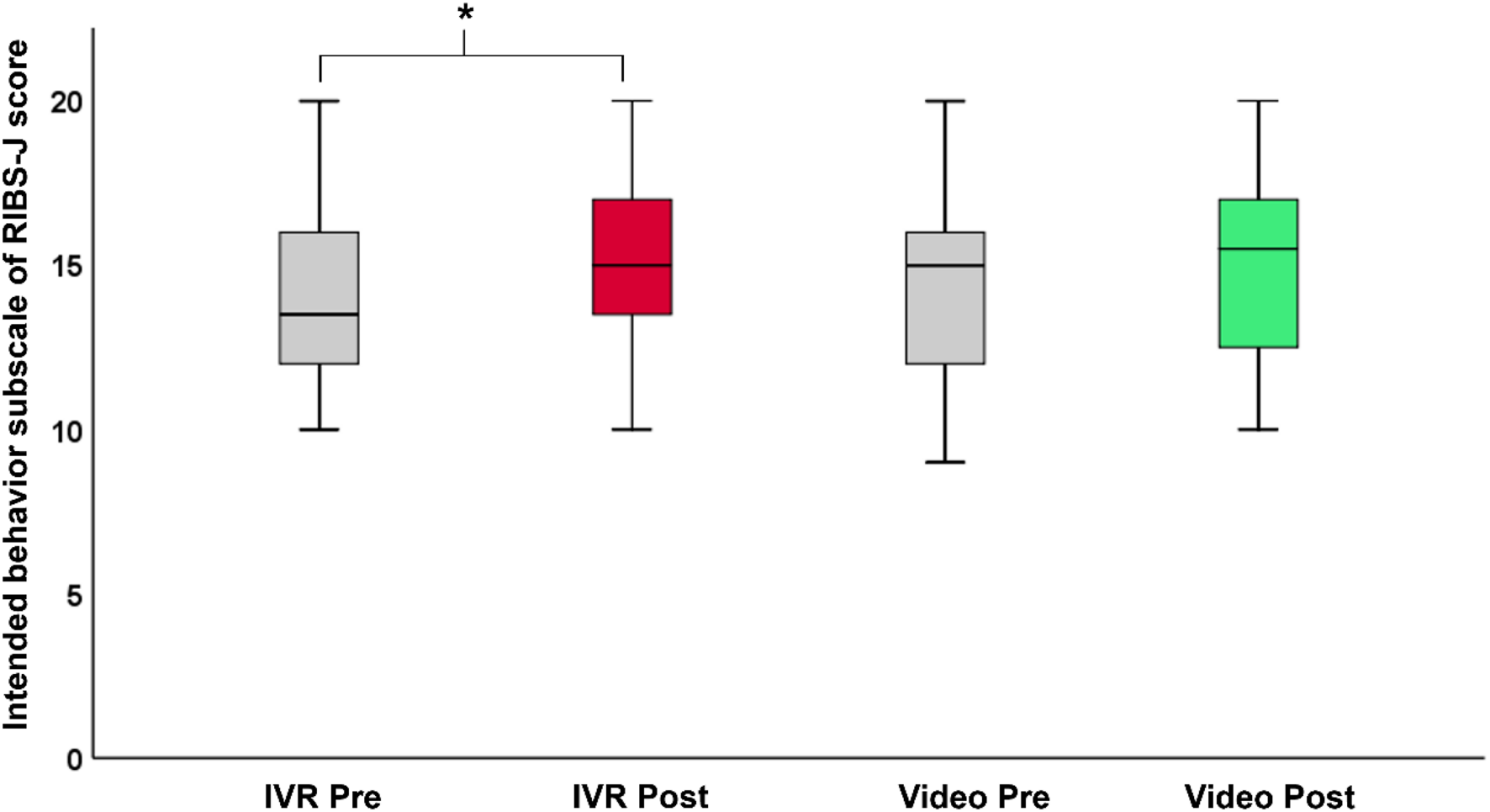
Change in the mean absolute total intended behavior subscale of RIBS-J score at pre- and post-interventions for the IVR and video. *IVR* immersive virtual reality, *RIBS*-*J* reported intended behavior scale—Japanese. *p < 0.05.

To evaluate the impact of anti-stigma interventions using IVR and video on stigma-related behavioral changes, we conducted a Friedman test across four-time points (pre- and post-intervention for both IVR and video). This analysis revealed a significant time effect on the intended behavior scores of RIBS-J (X^2^(3) = 9.86, *p* = 0.02). Subsequent post-hoc paired Wilcoxon signed-rank tests indicated a significant increase in the mean total score of the RIBS-J intended behavior subscale from pre- to post-interventions for the IVR intervention ([mean ± standard deviation] = 14.28 ± 2.99 vs. 15.41 ± 2.65, *p* = 0.01, adjusted for multiple comparisons using the Bonferroni method, where the p-value of 0.05 was divided by 4). In contrast, no significant difference was detected in video at pre- to post-interventions ([mean ± standard deviation] = 14.53 ± 3.01 vs. 15.16 ± 3.10, *p* = 0.05) (Fig. 3). There was also no significant difference between IVR and video interventions at pre-intervention (IVR vs. video, [mean ± standard deviation] = 14.28 ± 2.99 vs. 14.53 ± 3.01, *p* = 0.70) and at post-intervention (IVR vs. video, [mean ± standard deviation] = 15.41 ± 2.65 vs. 15.16 ± 3.10, *p* = 0.33).

### Association between depression knowledge-related changes and perception of re-experience of the intervention

A significant positive correlation was detected between the absolute change in the mean total MIDUS score and the perception of re-experience in the IVR intervention for thoughts related to the sense of personal experience (i.e. “I remembered the feeling as if it were my own experience”); that is, participants with a more significant absolute change in the mean total MIDUS score perceived the IVR intervention as a personal experience more vividly during the anti-stigma auditory fMRI task (Table 2).

**Table 2:** Correlation between the mean absolute total MIDUS score changes with the perception of re-experience of the first-person perspective anti-stigma intervention and total embodiment scores (n = 32).

	Change in MIDUS score			
	IVR intervention		Video intervention	
	*r*	*p*-value	*r*	*p*-value
Perception of the intervention				
Negative words	0.33	0.07	− 0.17	0.38
Positive words	0.11	0.55	0.04	0.83
Visual and not words	− 0.22	0.23	0.01	0.94
Neither visual nor words	0.02	0.93	0.19	0.29
Sense of being there	0.12	0.50	− 0.09	0.61
Sense of watching a movie	0.08	0.68	− 0.19	0.31
Sense of being a patient	0.19	0.29	− 0.22	0.23
Sense of depression	0.06	0.73	0.20	0.28
Sense of family/friend	0.09	0.64	− 0.28	0.12
Sense of personal experience	0.38	0.03*	− 0.20	0.26
Embodiment (total score)	0.15	0.42	− 0.38	0.03*

Pearson’s correlation coefficients (*r*) between the mean total MIDUS score changes with the perception of re-experience of the 1PP anti-stigma intervention sub-domains scores and total embodiment scores after IVR and video interventions. *MIDUS* mental illness and disorder understanding scale, *IVR* immersive virtual reality. *p < 0.05.

Further exploration of the data using paired t-test showed that there was a significantly higher score for IVR intervention compared to video intervention in four types of perception of re-experience of the intervention thoughts: the sense of being there (i.e. “I remembered the feeling as if I was there”), sense of being a patient (i.e. “I remembered the feeling as if I was the patient”), sense of depression (i.e. “I remembered the feeling as depression”), and sense of personal experience (i.e. “I remembered the feeling as if it were my own experience”) (Table 3).

**Table 3:** Paired-sample *t* test results on perception of re-experience of the first-person perspective anti-stigma intervention and embodiment scores (n = 32) [mean (SD)].

	IVR intervention	Video intervention	*p*-value
Perception of the intervention			
Negative words	3.16 (1.18)	2.81 (1.13)	0.11
Positive words	2.28 (1.07)	2.15 (0.94)	0.29
Visual and not words	4.15 (1.00)	4.03 (0.92)	0.47
Neither visual nor words	2.44 (1.17)	2.59 (0.86)	0.41
Sense of being there	3.78 (1.05)	2.71 (1.15)	0.01**
Sense of watching a movie	2.94 (1.09)	3.22 (1.17)	0.19
Sense of being a patient	2.75 (1.30)	2.06 (1.00)	0.01**
Sense of depression	2.59 (1.27)	2.13 (1.02)	0.03*
Sense of family/friend	2.09 (1.10)	2.38 (1.19)	0.18
Sense of personal experience	3.38 (1.24)	2.56 (1.14)	0.01**
Embodiment (total score)	0.95 (0.87)	− 0.99 (0.81)	0.01**

*SD* standard deviation, *IVR* immersive virtual reality.

*p < 0.05, **p < 0.001.

### Association between depression knowledge-related changes and embodiment

We observed a significant and negative correlation between the absolute change in the mean total MIDUS score and the total embodiment score for video intervention (Table 2). This result suggests that participants with more significant absolute changes in the mean total MIDUS score had lower total embodiment scores associated with video intervention. We observed a statistically significant difference when comparing the correlation coefficients between embodiment and MIDUS score changes across the IVR and video interventions (*z* = 2.1, *p* = 0.04), highlighting the distinctive impact of the immersive IVR experience on knowledge acquisition. In addition, the paired t-test showed a significantly higher embodiment score for IVR intervention than video intervention (Table 3).

### Effect of anti-stigma intervention-related brain activities on the change in depression knowledge

**Figure 4:**
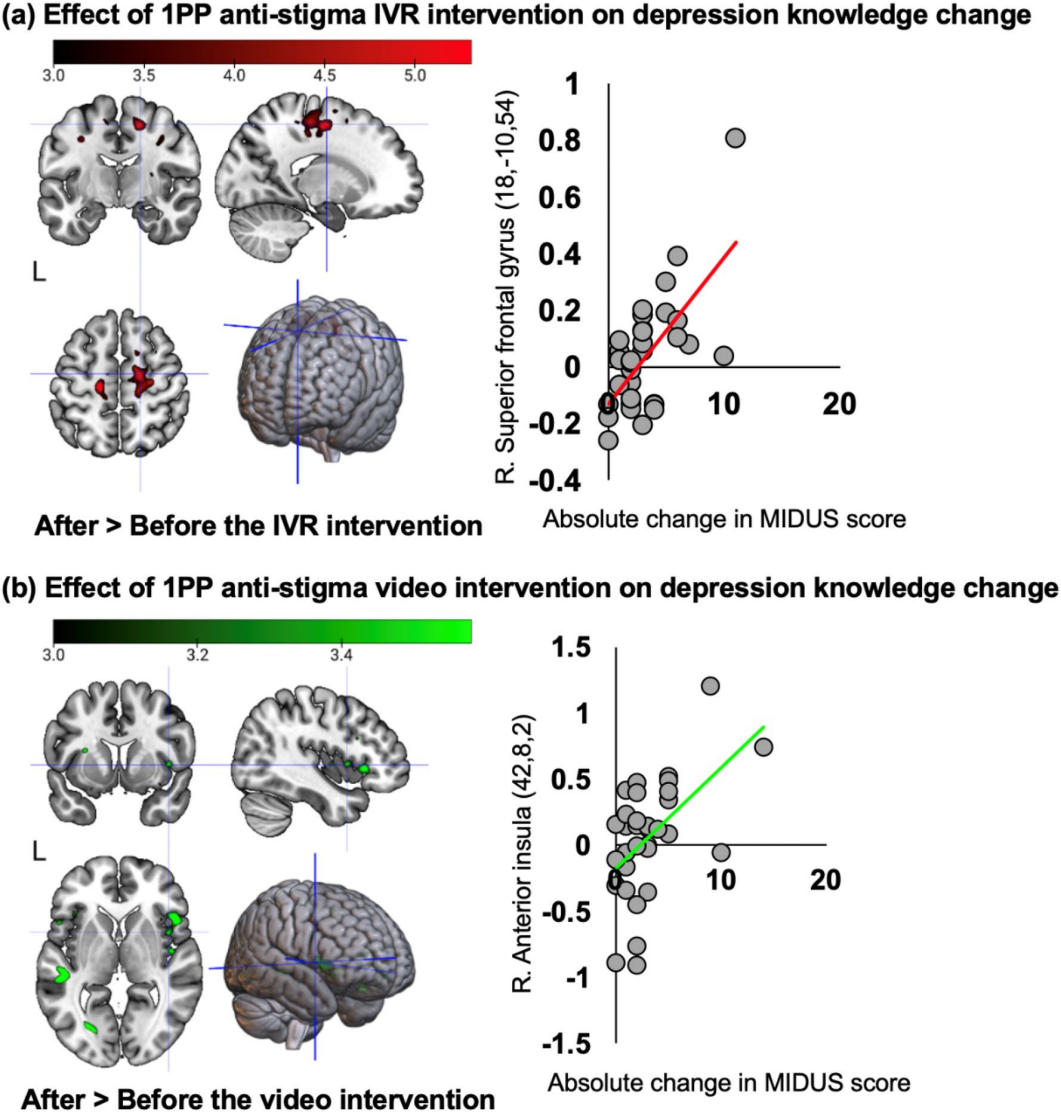
First-person perspective (1PP) anti-stigma intervention-related activation and the effect of the mean absolute total MIDUS score change. Red and green brain regions are positively active after the 1PP anti-stigma IVR and video interventions to the effect of the mean absolute total MIDUS response change, respectively. The activation profile for the (**a**) right superior frontal gyrus, and (**b**) right anterior insula represents the parameter estimates for each after > before intervention 1PP anti-stigma intervention-related activation (vertical axis) in each individual plotted against the significantly associated change in mean absolute total MIDUS score (horizontal axis). The coordinates in the MNI standard space and regression line are given for each plot. *IVR* immersive virtual reality, *MIDUS* mental illness and disorder understanding scale.

The results of the voxel-wise regression analyses on the absolute difference between pre-and post-interventions of the mean total MIDUS score with anti-stigma intervention-related brain activation for both IVR and video interventions are summarized in Table 4 and Fig. 4. Concerning the IVR post-intervention (i.e. after > before the IVR intervention), a significant effect of mean total MIDUS score absolute change was identified in the right SFG, bilateral PrG, and left SMC. For the video post-intervention (i.e. after > before the video intervention), a significant positive effect of mean total MIDUS score absolute change was identified in the right anterior insula and frontal operculum. Neither significant IVR pre-intervention activation (i.e. after < before the IVR intervention) nor video pre-intervention activation (i.e. after < before the video intervention) was identified. In addition, the observed positive effects in the brain regions mentioned above were not replicated in the video intervention at a liberal statistical threshold, and vice-versa.

**Table 4:** The significant effect of IVR and video interventions on the absolute change in mean total MIDUS score.

Brain region	Peak			t	Cluster	
	x	y	z		k	p
After > before the IVR intervention						
R. superior frontal gyrus	18	− 10	54	5.31	985	< 0.001
R. precentral gyrus	28	− 16	56	4.99		
L. precentral gyrus	− 12	− 22	64	5.32	437	0.001
L. supplementary motor cortex	− 10	0	62	4.34		
After < before the IVR intervention						
n.s						
After > before the video intervention						
R. anterior insula	42	8	2	3.58	388	0.001
R. frontal operculum	46	22	0	4.70		
After < before the video intervention						
n.s						

The coordinates (x, y, z) and the *t*-value of peak activation, the size and *p*-value of the clusters are given for after > before and after < before the first-person perspective anti-stigma auditory fMRI task for depression knowledge acquisition-related brain activations in the IVR and video interventions (i.e. regression analysis).

*IVR* immersive virtual reality, *MIDUS* mental illness and disorder understanding scale, *L* left, *R* right, *n.s* not significant.

## Discussion

This study explored the effects of 1PP anti-stigma interventions using IVR and video on depression knowledge, stigma reduction, and the corresponding depression knowledge brain activity change assessed by fMRI. Participants engaged in an IVR intervention and viewed similar content on a video monitor, which included the daily routine of a patient with mild depression through scenes on depression and recovery. While both interventions improved depression knowledge, only the IVR intervention effectively reduced stigma. Depression knowledge score was positively associated with neural response to the IVR intervention in the right SFG activation, implicated in empathic concern. On the other hand, depression knowledge scores positively with the neural responses related to video intervention in the right anterior insula, implicated in distress-related response. As discussed below, the 1PP anti-stigma IVR intervention differs from the video intervention in terms of its effectiveness in reducing stigma and the brain regions related to depression knowledge score, in which IVR benefits from by increasing sense of presence and embodiment towards patient with depression.

Depression knowledge improved in the IVR and video interventions and might be attributed to the 1PP anti-stigma intervention contents. In the current intervention, the contents included patient testimonials in 1PP, stories emphasizing recovery and avoiding the use of excessive information. Previous studies showed that the usage of testimonies narrated from the 1PP^8,40^, a story emphasizing on recovery and/or the possibility of living a good life despite the mental illness^8,15^, and reducing cognitive overload^21^ can contribute to a convincing storytelling experience, enhancing learning and knowledge gain^41^.

There was a significant stigma reduction only in the IVR intervention and may have been due to the immersive experience. The sense of being in the body of a patient with depression, which was the most remarkable feature of the IVR experience^17–19^, was associated with more realistic behavioral responses compared to the same environment in video^13^. This immersive experience enabled participants to ‘walk in the shoes’ of a patient with depression and this may have successfully convinced them on the importance to reduce the stigma^42^. Conversely, stigma was not significantly reduced after the video intervention. The change in depression knowledge for the video intervention was negatively associated with embodiment, suggesting that participants may have not acquired the depression knowledge adequately from the patient’s perspective due to the low synchronization. We speculated that the depression knowledge improved in the video intervention may have reflected the accumulation of negative knowledge about patients with depression. It has been suggested that the understanding of suffering gained through distress-related response does not necessarily lead to prosocial behavior but may lead to a desire to withdraw from interaction as a means to protect oneself^43^. Thus, this desire to protect oneself may have mitigated the effectiveness of video intervention in reducing stigma.

Our findings from the IVR intervention for depression knowledge align with the notion that the acquired knowledge fosters empathic concern. Specifically, we observed that an increase in depression knowledge scores was closely linked to participants reported personal experience during the immersive re-experience of the 1PP anti-stigma IVR intervention. Furthermore, this increase in knowledge was positively correlated with activation in the R.SFG, a brain region previously associated with empathic concern towards others, especially those perceived as being in out-groups or physically ill^27,31^, and regarded as a stimulus with positive emotional valence. We measured individual differences by examining the absolute change in MIDUS scores, revealing that individuals showing greater improvements in depression knowledge also exhibited increased R.SFG activation. This finding complements previous research indicating that higher R.SFG activation occurs in individuals working to overcome negative implicit biases towards stigmatized groups, suggesting a heightened cognitive effort for empathy^32^. Additionally, a related study identified a link between social dominance—a factor associated with stigma—and activation in the left SFG^44^, further supporting our interpretation that significant knowledge gains might require increased empathic cognitive effort. Specifically, this study^44^ observed greater left SFG activity among participants who were higher on social dominance orientation and, thus, were likely to be more sensitive to the contempt expressed by others, which, in turn, may have induced stronger emotion regulation efforts. Therefore, we propose that the positive correlation between the change in depression knowledge and R.SFG activity implies that individuals who learn more about depression through our IVR intervention are applying greater cognitive effort toward understanding and empathy. This enhanced knowledge gain is likely facilitated by the vivid and realistic simulation of experiencing mild depression symptoms, encountering public stigma, and receiving support within a multisensory perceptual IVR environment, highlighting the intervention's effectiveness in promoting empathic concern.

The neural finding for depression knowledge using video intervention agrees with the view that this acquired knowledge reflects an individual tendency for distress-related response. A positive correlation was identified in the right anterior insula previously implicated in racial prejudice experience^28^ and empathic distress felt towards mentally ill patients^31^ and considered as negative valence. Specifically in terms of individual variation, negative explicit attitudes toward homeless individuals^33^, social exclusion experience^24^, individual’s disgust sensitivity^28^ were associated with increased activity in the anterior insula. Our findings suggest that depression knowledge acquired using anti-stigma video intervention may reflect inefficiency in the knowledge improvement, which can be interpreted in the context of unfavorable social interaction.

We speculated that the difference in right SFG and right anterior insula brain activation patterns between 1PP anti-stigma IVR and video interventions may be due to a higher score in the sense of presence and sense of embodiment felt in the IVR intervention. This explanation may be supported by the comparison results of the four types of perception of re-experience intervention thoughts (i.e. the sense of being there, sense of being a patient, sense of depression, and sense of personal experience) and embodiment total score. Participants felt more sense of presence (i.e. the illusion of “being there”) and synchronized movement with the virtual avatar hand in the IVR intervention (i.e. embodiment) than in the video intervention. This confirmed that our 1PP anti-stigma IVR intervention approach led to a greater sense of presence and embodiment in the virtual environment. Thus, a higher sense of presence and synchronized movement with the virtual avatar hand means that the 1PP anti-stigma IVR intervention might have contributed to forming depression knowledge through neural mechanisms different from the video environment.

Although this study offers novel insights, particularly the relationship between the right SFG and the effect of 1PP anti-stigma IVR intervention using a combination of cross-over RCT and fMRI, it is not without limitations. First, the duration between the first and the second interventions varied between participants with a minimum of 1 day to a maximum of 49 days (average 11.75 days). Although the carryover effect was insignificant, applying longer intervals between one type of intervention and the other would be better. However, considering the availability of participants to participate on two different days and the availability of the MRI schedule, we assumed that longer periods would increase the number of withdrawals from participation in our experiment. Second, we should have investigated whether the effect of the IVR intervention persists over a long period and if the IVR intervention is effective in changing participants' behavior in real life. Third, mixed findings may be due to the lack of significant difference in stigma-related behavioral changes between IVR and video in post-interventions. Fourth, as it is impossible to carry out IVR intervention during the MRI, we prepared the perception of re-experience of the 1PP anti-stigma intervention questionnaire to verify if the participants could relive their experience of the IVR and video interventions within the MRI scanner. Although some of the questions were similar to those in the embodiment questionnaire, the perception of re-experience of the intervention questionnaire was not validated, and further validation may be needed. Last, the limitations of the current IVR technology in fully expressing the subtleties of depression symptoms could have impacted the effectiveness of this study.

In conclusion, our findings indicate that while both IVR and video interventions enhanced depression knowledge, only the immersive nature of the IVR intervention led to a significant reduction in stigma. This was facilitated by increased activation in brain regions associated with empathic concern, highlighting the potential of immersive technologies in stigma reduction initiatives.

## Methods

### Ethical approval

All participants provided written informed consent to participate in this study and were monetarily compensated for their participation. This study was approved by the Research Ethics Committee of the Faculty of Medicine and Graduate School of Medicine of the University of Tokyo (2019099NI) and conducted according to the Declaration of Helsinki.

### Study design

This was a crossover RCT (Refer to Supplementary Figure S1). The crossover RCT was chosen because it allows the participants’ response in IVR intervention to be contrasted with the same participant's response in video intervention. According to a previous study^45^, removing participant variation through the crossover RCT is potentially more efficient than similar-sized, parallel-group trials, where each participant was exposed to only one intervention. Participants were cross-over randomized 1:1 to start with one of the two types of intervention with the same anti-stigma intervention content: (1) an interactive IVR intervention using a head-mounted display (HMD) (Fig. 1a), or (2) a non-immersive and non-interactive video intervention in the form of a computer screen. All participants were required to participate in the 2-day intervention study. In other words, participants who underwent IVR intervention on the first measurement day (Day 1) and after a washout period (1–49 days), underwent video intervention on the second measurement day (Day 2), and vice-versa.

The randomization was performed by randomly allocating a number from 0 to 1 to each participant using Microsoft Excel software. Randomization, enrollment, and assignment of participants were performed by the main experimenter overseeing the trial. Neither the participants nor experimenters were blinded in this study. This study adhered to the CONSORT guidelines^46^ (Refer to Supplementary Figure S1) and was registered at the University Hospital Medical Information Network Clinical Trials Registry (UMIN-CTR) (Identifier: UMIN000043020, First registration date: 20/01/2021), Japan.

### Participants

Of the 40 participants assessed for eligibility, 36 (13 female; age range = 19–46 years, age [mean ± standard deviation] = 24.00 ± 6.10 years; Table 1) were recruited from the undergraduate and graduate schools of The University of Tokyo and social media advertisements between November 2022 and February 2023. The inclusion criteria consisted of being a native speaker of Japanese. Participants were excluded if they were left-handed or reported any of the following conditions: a history of psychiatric/neurological diseases, pregnancy, claustrophobia, or MRI contraindicated implants. The primary outcome was behavioral data. We determined the sample size according to a previous study, which used IVR technology to reduce stigma^40^; the sample size was calculated using G*Power 3.1.9.7 software to allow performance of a repeated measure ANOVA with a significance level (α) of 5%, power of 80%, and effect size of 0.44 (Cohen’s d), which yielded a recommended sample size of 30 participants. To avoid the possibility that some of the data may be unusable due to head movement or other uncontrollable reasons, we enrolled 40 participants.

### Intervention

#### Apparatus

The IVR environment was presented via an HMD (‘Oculus Quest 2’; 120 Hz refresh rate, 1832 × 1920 resolution per eye, 104° field of view horizontal) with active noise cancellation intra-auricular earphones (Bose QC 20, USA) and hand tracking was used as a means of control in the virtual environment. It was developed using Unity3D (Unity Technologies, San Francisco, CA) game engine and the computer-aided design (CAD) software Blender. The programming language chosen was C#. The software for creating the virtual characters was VRoid Studio v1.11.1, a hub of freely available 3D anime-based models.

The equipment used for the video intervention was a laptop (Windows OS 10, refresh rate of 60 Hz, 1920 × 1080 non-interlaced monitor resolution) with active noise cancellation intra-auricular earphones (Bose QC 20, USA). The laptop was positioned below the participant’s eye level, and the approximate distance from the screen to their eyes was about 45 cm (visual angle of about 42 degrees). The video intervention was presented using Windows Media Player. The content of the video intervention was the same as the IVR intervention (Fig. 1b); however, no interactions were required from the participants while watching the video.

#### 1PP anti-stigma intervention task

**Figure 5:**
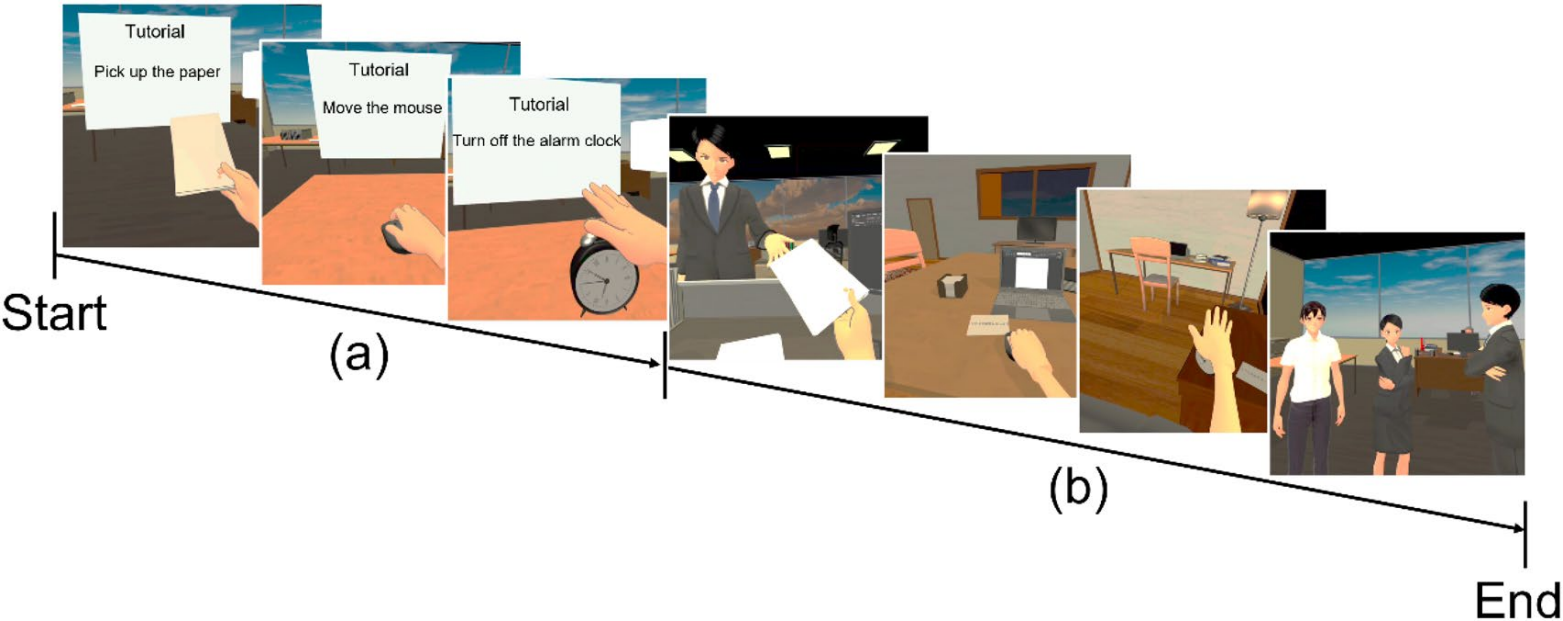
Progression screenshot of the first-person perspective (1PP) anti-stigma intervention content. (**a**) Tutorial phase: the participant was familiarized with the activities that will be carried out in the main scenario (i.e. picking up the paper, moving the mouse, and turning off the alarm clock), and a brief description of the public stigma was also provided, (**b**) Main scenario: the participant assumed the role of a company employee with mild symptoms of depression in the 1PP and experienced public stigma by two co-workers, and the support from a colleague at the same company and his/her mother.

The IVR and video intervention contents used were adapted from our 1PP anti-stigma application^47^. The 1PP anti-stigma intervention task consisted of two phases: tutorial (Fig. 5a) and main scenario (Fig. 5b). In the tutorial phase, the participant was familiarized with the activities to be carried out in the main scenario (i.e. picking up the paper, moving the mouse, turning off the clock), and a brief description of the public stigma was provided before proceeding. In the main scenario phase, the participant assumed the role of a company employee with mild symptoms of depression^48^ in the 1PP, experienced public stigma from two co-workers, and also support from a colleague and his/her mother. In terms of mild symptoms of depression, the participants were instructed to experience symptoms portrayed in IVR, such as inability to concentrate at work, loss of interest in previously interesting activities (anhedonia), sleep deprivation (insomnia), loss of appetite, tiredness, and inability to move (lethargic). Regarding the public stigma experience, the participant experienced stigmatized attitudes from two co-workers such as discrimination (i.e. shunning the patient with depression), prejudice (i.e. reluctant feeling to be in the same office as the patient with depression), and stereotypes (i.e. wrong understanding that depression is a reason for malingering). The scene on recovery from depression and the return to work was shown to promote the understanding that a patient with mild symptoms of depression can recover with proper treatment and care from friends and family who support his/her recovery.

### Assessments

#### Demographic characteristics

We obtained the demographic characteristics of the participants using a pre-intervention behavioral assessment. The demographic characteristics were, age, sex (female/male), previous contact with depression patients, learning about depression, and previous IVR experience. Regarding previous contact with depression and learning about depression, these were included as a demographic characteristic because the information has known to affect knowledge of depression in the Japanese population^36^. Except for age and sex, participants were asked to rate their responses by simple yes/no decision units on a binary scale.

#### Depression knowledge-related behavioral changes

To assess depression knowledge-related behavioral change, we employed the Mental Illness and Disorder Understanding Scale (MIDUS). The MIDUS assessed the participants’ accurate knowledge of mental illness^36^ (e.g. ‘mental illness is treatable’, ‘mental illnesses are common’). The MIDUS consisted of 15 items rated on a 5-point Likert scale (0, *strongly agree*; 4, *strongly disagree*; range, 0–60). The MIDUS scale was developed in Japan and has good internal consistency and validity^36^. We used the mean total MIDUS score, as in the previous survey^36^. A low total score indicates a good understanding of accurate knowledge of mental illness. In addition, for the fMRI analysis and other questionnaire associations, the change in mean total MIDUS score was defined using the absolute difference between the total mean MIDUS score^36^ for each pre- and post-interventions of the IVR and video. The Cronbach’s α was 0.78 in the Japanese population^36^. In the current dataset, Cronbach’s α was 0.68 for the IVR and 0.82 for the video in the pre-interventions.

#### Stigma-related behavioral changes

To measure individual tendencies of respondent’s future behavioral intentions towards patients with depression, we used the intended behavior subscale of the RIBS-J^37^. The intended behavior subscale consisted of 4 items rated on a 5-point Likert scale (1, *strongly disagree*; 5, *strongly agree*; range, 4–20). The RIBS-J was created based on the original RIBS, which was developed in the UK^38^ and has good internal consistency and validity for the Japanese population^37^. The total mean score of the intended behavior subscale was used, similar to that in the previous study^37^. Higher scores on the intended behavior subscale indicate more positive intentions towards the patient with mild depression. The Cronbach’s α was 0.71 in the Japanese population^37^. In the current dataset, Cronbach’s α was 0.79 for the IVR and 0.84 for the video in the pre-interventions.

#### Perception of re-experience of the 1PP anti-stigma intervention measure

Participants were asked to intuitively respond to how they relived the 1PP anti-stigma intervention inside the MRI scanner. We prepared ten questions rated on a 7-point Likert scale (1, *strongly disagree*; 7, *strongly agree*). The questions were related to negative words (“I remembered that the feeling is similar to words like “sorrow”, “suffering”, etc.”), positive words (“I remembered that feeling similar to the words “joy”, “happiness”, etc.”), visual and not words (“I remembered that feeling similar to the sense of sight and not words”), neither visual nor words (“The feeling cannot be expressed using the sense of sight nor words”), sense of being there (“I remembered the feeling as if I was there”), sense of watching a movie (“I remembered the feeling as if I was watching a movie”), sense of being a patient (“I remembered the feeling as if I was the patient”), sense of depression (“I remembered the feeling as depression”), sense of family/friend (“I remembered the feeling as if it was a family member or friend that was dear to me”), and sense of personal experience (“I remembered the feeling as if it were my own experience”). Each item was rated independently by the participants.

#### Embodiment measure

Evaluation on the embodiment of the IVR and video interventions were assessed using a 14 items embodiment questionnaire rated on a 7-Likert scale (-3, *strongly disagree*; 3, *strongly agree*). The questionnaire was adopted from previous studies on the embodiment of virtual avatar^39,49^ and prepared to ask if the distinction was made between the participant’s arm and the virtual arm. The total embodiment score was calculated based on the original study^39^, excluding questions not assessed in this study. In our dataset, Cronbach’s alpha was 0.81 for the IVR and 0.85 for the video post-interventions.

### 1PP anti-stigma auditory fMRI task

In addition to the 1PP anti-stigma intervention task, each participant performed a 1PP anti-stigma auditory fMRI task to assess the association between the absolute difference in the pre- and post-interventions of MIDUS score with brain activation change of IVR and video interventions. The instructions and 1PP anti-stigma auditory fMRI task (Fig. 5b) without visual information were presented via MRI-compatible headphones (SS3300; Avotec, Inc., Stuart, FL). In each block, an instruction for assigning the task was presented aurally for 6 s. The 1PP anti-stigma auditory fMRI task was sounded for 240 s, during which the participant was asked to listen to the audio stimulus and imagine themselves in the situation, followed by a rest of 15 s. Four blocks were performed over 19 min for each pre- and post-interventions. The 1PP anti-stigma auditory fMRI task stimulus was presented using PsychoPy v2021.2.3^50,51^.

### Overview of experiment procedures

The experiment was carried out for 2 days in two interventions (i.e. pre- and post-interventions). Participants answered demographic characteristics, MIDUS, and intended behavior subscale at the beginning of the pre-interventions. At the end of the post-intervention, they answered another set of MIDUS, intended behavior subscales, questions on the perception of re-experience of the 1PP anti-stigma intervention and embodiment questionnaire. In the pre- and post-intervention, participants underwent two MRI scanning (i.e. pre- and post-interventions) with 1PP anti-stigma auditory task for both types of interventions (i.e. IVR or video interventions). During the MRI scanning, participants were laid in the MRI scanner with foam pads and a headband to minimize head motion. In addition, they entered the MRI scanner with a sleeping mask over their eyes to minimize the visual effects generated on removing the HMD after the IVR intervention. They listened to the instruction and 1PP anti-stigma auditory fMRI task via MRI headphones.

After the pre-intervention of the fMRI scanning, participants were given approximately 15 min of rest between interventions (i.e. between pre- and post-interventions) before carrying out the intervention (i.e. IVR or video interventions). For the IVR intervention, participants were seated on a 360-degree rotation chair, where the intervention was the 1PP anti-stigma intervention presented via HMD. The participants were then briefed on the use of the HMD by the experimenter. Participants underwent the IVR intervention after setting up the HMD with active noise cancellation intra-auricular earphones. The IVR intervention began when participants placed their hands located a few centimeters in front of the midline of their body, at position [0, 0, 0] on the XYZ plane of the virtual environment, for the HMD to detect their hand. Participants were instructed to stop the intervention if they experienced any IVR sickness (similar to motion sickness) during the intervention. At the end of the IVR intervention, participants were told to close their eyes prior to removing the HMD and were blindfolded after taking off the HMD.

The video intervention was conducted through a laptop screen where the participants were seated on a 360-degree rotation chair with active noise cancellation intra-auricular earphones. Participants were instructed to start the video using the computer mouse. The video intervention began when participants clicked on the ‘play button’ on the video on the computer screen.

After both interventions, the blindfolded participants were guided to the MRI scanning room by the experimenter for the post-intervention fMRI scanning. Similar to the fMRI pre-intervention, participants used a sleeping mask over their eyes and were instructed to listen to the audio stimulus during the fMRI scanning and imagine themselves in the situation. The structural brain image was taken after the 1PP anti-stigma auditory fMRI task video post-intervention for each participant.

### MRI data acquisition

All fMRI data were acquired with a 3-T whole-body MRI system (MAGNETOM Prisma; Siemens, Erlangen, Germany) with a 64-channel head coil. In the 1PP anti-stigma auditory fMRI task, we acquired 39 continuous slices of functional images per volume (field of view (FOV) = 192 × 192 mm^2^, 64 × 64 matrix, slice thickness = 3 mm, slice gap = 0.75 mm) with an echo planar imaging (EPI) pulse sequence (repetition time (TR) = 2000 ms, echo time (TE) = 25 ms, flip angle (FA) = 90°). We acquired 570 volumes during the pre- and post-interventions. All slices were tilted by 30° from the anterior/posterior commissure plane to the forehead. We also acquired whole-brain high-resolution T1-weighted images (1 × 1 × 1 mm^3^) with a magnetization-prepared rapid acquisition gradient echo sequence (TR = 2000 ms, TE = 2.9 ms, FA = 9°; FOV = 256 × 256 × 256 mm^3^). We visually inspected all structural and functional images to assess image quality and movement artifacts. In addition, we instructed all participants to relax and not to fall asleep.

### Analysis

#### Behavioral data analysis

We compared demographic characteristics between the two sequences of intervention types (IVR-video or video-IVR sequences) using *t*-tests for continuous variables and chi-square tests for categorical variables.

The Shapiro–Wilk test was used to confirm the normality of the MIDUS and intended behavior subscale of IVR and video at the pre-intervention (MIDUS: IVR intervention, *p* = 0.50; video intervention, *p* = 0.40; intended behavior subscale: IVR intervention, *p* = 0.01; video intervention, *p* = 0.49) and post-intervention (MIDUS: IVR intervention, *p* = 0.10; video intervention, *p* = 0.44; intended behavior subscale: IVR intervention, *p* = 0.18; video intervention, *p* = 0.07). There was a normal and no normal distribution by the Shapiro–Wilk test for the MIDUS and intended behavior subscale, respectively. In addition, to identify possible carry-over effects^52,53^ attributable to the cross-over design, the differences in the MIDUS and intended behavior subscale between the IVR-video and Video-IVR sequences were investigated using the t-test and Wilcoxon signed-rank test, respectively. Two-way RM-ANOVA and Friedman tests were conducted for MIDUS and intended behavior subscale, respectively, with time as a within-subject factor (pre-intervention vs. post-intervention) and intervention type as the between-subject factor (IVR intervention vs. video intervention). For post hoc analysis after two-way RM-ANOVA, we carried out multiple comparisons using the Bonferroni correction. For post hoc analysis after the Friedman test, we carried out the Wilcoxon signed-rank test, and used the Bonferroni corrected *p*-value.

Person’s correlation tests were performed to examine the correlations between the absolute change in MIDUS scores in the IVR and video interventions with the perception of re-experience on the 1PP intervention and the total embodiment scores. To compare the embodiment scores' correlation coefficients between the IVR and video interventions, we employed Fisher's z-transformation, elucidating the significant distinction between the two modalities in facilitating depression knowledge through embodiment. In addition, a paired-sample *t*-test was performed to probe the difference in each subdomain of the perception of re-experience on the 1PP intervention and total embodiment scores between IVR and video interventions.

Statistical analysis was performed using the statistical package for the social sciences (SPSS) software version 25 (IBM SPSS Inc. Chicago, IL) for Windows, and statistical significance was set at *p* < 0.05.

#### fMRI data analysis

We conducted preprocessing using statistical parametric mapping software (SPM12; Wellcome Department of Imaging Neuroscience; Institute of Neurology, London, United Kingdom) implemented on MATLAB R2017b (MathWorks Inc., Natick, MA, USA). Preprocessing included correction for head motion, co-registration to anatomical image, spatial normalization using the anatomical image and the Montreal Neurological Institute template, and smoothing using a Gaussian kernel with full width at a half-maximum of 6 mm. None of the registrations of the participants’ images differed between the pre- and post-interventions.

A conventional two-level fMRI analysis was adopted using SPM12^54,55^. As a first-level analysis, the degree of activation or deactivation from the 1PP anti-stigma auditory fMRI task was estimated for each participant using a general linear model (GLM). Pre-processed images of the pre- and post-interventions and two hemodynamic models (i.e. after < before the IVR intervention, after > before the IVR intervention, after < before the video intervention, and after > before the video intervention contrasts) were constructed using the standard hemodynamic function supplied by SPM12. Six estimated head motion parameters were included in the GLMs as confounding factors.

At the second-level analysis, the neural correlates for the absolute changes in the MIDUS scores were estimated using a voxel-by-voxel multiple regression analysis of 1PP anti-stigma auditory fMRI task responses across the participants for IVR and video interventions. For each positive and negative 1PP anti-stigma auditory fMRI task response, the absolute difference between the total mean MIDUS score for each pre- and post-interventions of the IVR and video interventions were included as an independent variable of interest. In contrast, age and sex were included as covariates. After that, the effect of the 1PP anti-stigma auditory fMRI task response at the peak voxels of the identified activation clusters for IVR intervention was verified for the 1PP anti-stigma auditory fMRI task video response, and vice-versa using a liberal statistical threshold for the region-of-interest (ROI) analysis. The ROI approach verifies whether the activation peaks observed in IVR interventions are also observed for the video intervention and vice-versa. The voxel-wise statistics used an uncorrected *p* < 0.005 for the cluster-forming threshold, set at a family-wise error-corrected *p* < 0.05 for cluster extent. The statistical threshold for the ROI analyses was set to an uncorrected p < 0.05. The identified brain structures were anatomically labeled using the SPM Anatomy toolbox^56^.

### Supplementary Information

Supplementary Figure S1.

## Data Availability

Data obtained in the current study will be available from the first authors (W.G.L. and K.H.d.S.K.) upon reasonable request, including a project outline.
