# Supplementary material for: Exploring the impact of immersive virtual reality on depression knowledge and stigma reduction: a cross-over RCT fMRI study
Source: Sci Rep. 2024 Mar 2;14:5193. doi: 10.1038/s41598-024-55797-w (PMC10908822; doi:10.1038/s41598-024-55797-w)
Supplement: Supplementary file 1 — Supplementary Figure S1. [file 41598_2024_55797_MOESM1_ESM.docx]

**Supplementary Figure for**

Exploring the Impact of Immersive Virtual Reality on Depression Knowledge and Stigma Reduction: A Cross-Over RCT fMRI Study

Wey Guan Lem^1*‡^, Kelssy Hitomi dos Santos Kawata^1‡^, Hiroshi Oyama^1^

^1^Department of Clinical Information Engineering, Graduate School of Medicine, The University of Tokyo, Hongo 7-3-1, Bunkyo-ku, Tokyo, 113-0033, Japan.

^‡^These authors contributed equally to this work

**Supplementary Figure and Figure Legend**





**Supplementary Fig. S1** CONSORT flow diagram showing the procedural flow of the cross-over RCT fMRI study.
